# An Integrated Multi-Sensor Network for Adaptive Grasping of Fragile Fruits: Design and Feasibility Tests

**DOI:** 10.3390/s20174973

**Published:** 2020-09-02

**Authors:** Yuanxin Xie, Baohua Zhang, Jun Zhou, Yuhao Bai, Meng Zhang

**Affiliations:** College of Artificial Intelligence, Nanjing Agricultural University, Nanjing 210031, China; 32317421@njau.edu.cn (Y.X.); zhoujun@njau.edu.cn (J.Z.); 2020112041@njau.edu.cn (Y.B.); 32117128@njau.edu.cn (M.Z.)

**Keywords:** agricultural robot, gripper design, multi-sensor network, adaptive grasping

## Abstract

Secure grasping of fragile fruits and other agricultural products without potential slip and damage is still a challenge due to the size and shape varying, bruise susceptible, as well as hardness changing during fruit and vegetable maturation. In the robotic grasping process, the mechanical damage mainly depends upon the aggressiveness of the gripper and the sensitivity of the product to the damage. In this study, a flexible gripper integrated with multi-sensor network is designed and tested. The network proposed includes three kinds of sensors that enable the gripper to grasp various products with the sense of touch and visual perception. Particular attention has been attached to the sensors applied between the fingers, and this makes sensing and grasping capabilities improved. To create an accurate grasping system, a grasping algorithm and the force control model are proposed for any bending state based on Cosserat theory. The boundary detection is included in the grasping algorithm, detecting the shape edge by some certain point calculation. The created grasping system guarantees mechanical compliance by evaluating and adjusting the finger status including force, angle, and direction. Multi-group tests have been done on grasping several objects of different sizes and materials in daily life. The relationship between force, bending, and surface material is also analyzed and compared under different conditions. The numerical comparisons related to the measurement error are analyzed based on their standard deviations. Experimental results indicate that this flexible manipulator with proposed system and strategy has better grasping ability for fragile fruits with its good flexibility and dexterity.

## 1. Introduction

The gripper, one of the key parts of mechanical equipment and the final capabilities of the machine, is directly determined by its manufacturing level. Robotic grippers are widely applied in agriculture and industrial manufacturing, due to the properties and sizes of agricultural products varied and the frequent changes in industrial materials [1]. Human perception of the outside world is facilitated by the success of autonomous operation, and huge demands are satisfied through direct contact between the robot and the surrounding targets [2]. As a tool for grasping and operating objects, the damage must be minimized during the grasping and moving process [3,4]. Higher requirements are proposed for the robot end effector to act as the interface of data acquisition and information perception. Therefore, studying the flexibility and perception ability of manipulator would be useful.

Robotic grippers, being used in agriculture, help replace or supplement humans to perform some tedious or repetitive tasks such as picking and placing fruits or vegetables [5]. As new human–environment interaction tools, an extensive application space is provided by them [6]. Compared with industrial manipulators, an insight review [7] noted that softness and body compliance have been applied to agricultural grippers as essential features for its flexibility, and this objective has been reached with the combination of special materials, under actuated mechanisms, and a bioinspired design [5,8]. In addition, some work has been done using perceptive techniques [9,10] to identify the objects during post-working inspection and operations. In some cases, the optimum picking technique recommended by both Yang et al. [11] and Du et al. [12] requires sensory knowledge of the object’s orientation and target’s location. Therefore, ensured adaptability and improved flexibility are shown in their interactions with the environment.

Grasping, as an activity with strong interactive properties, is the main interface for the robots’ communication with another object. The traditional robotic grippers are mostly rigid structures when precise and rigid grasping is primarily considered in industrial process [13]. However, since the manipulating targets in agriculture are often delicate, fragile, and easily bruised fruits or vegetables, and highly varied in sizes, shapes, and materials [3]. Therefore, the flexible gripper has been increasingly examined. To improve the flexibility of robotic grippers, previous research illustrated the soft gripper concept where the fingers are changed into special materials like soft silicone materials [14,15,16]. For rigid or difficult grasping of fragile or irregular objects, it can be safely resolved by a soft gripper [14,17]. However, due to the problem of insufficient transverse stiffness, uncertainties still exist when it faces some heavy objects. To fix such deficiency, the application of pneumatic manipulator has been widely introduced [18], grasping the objects by activating a powerful inward suction force within the suction cups, which enables the movement of the object [19]. Nevertheless, this full open-close tool is not suitable for the installation of sensors, as while the whole process can be simply described as the charge and the release of the gases, the intermediate process is difficult to control with a lack of gripping dexterity as well [20]. More research is needed for the manipulator with good flexibility and excellent adaptability [21]. Since the agricultural machinery operations and sequence of robotic motions are even entirely different from one task to another, higher demands are raised to the flexibility of the manipulator design.

In order to accomplish a grasping task, the target should be perceived precisely first [22]. The intelligent manipulator, a tool for the robot to grab the object and operate the target, is also an interface for data acquisition and perception [23]. With the continuous improvement of sensor technology, a variety of sensors are inevitably proposed and used. The information of the environment can be gathered by the vision sensors for further object localization and object edge extraction [12]. In addition, bending sensors, detecting the position with a variable resistor that responds to bending, provides an effective way to control the bending angle. The measurement of grasping conditions is enabled with the help of single tactile sensor in fingers or fingertips, and it can be applied for real-time precisely controlling of the grippers [3]. A closed loop-controlled grasping system with the use of a dynamic pressure sensor is also applied in this adaptive gripper. Based on the piezo resistive effect, the micro pressure sensor is used [24]. In order to imitate the advantage aptitudes of human hands, multi-sensor network is established for the sense of touch and visual perception [25]. The precise use of multiple sensors will be converted into real-time sensory feedback to realize the grasping close-loop control to achieve grasping detection. Considering the information acquisition process, the installed sensor will collect the feedback signal from the information processing layer [14]. Since the manipulating targets are fragile, delicate, and easily bruised fruits, sensors in combination with the control technology makes the operation of the grippers more intelligent.

The specific target of this study includes investigating adaptive grasping of fragile fruits using a flexible gripper with an integrated multi-sensor network. In order to successfully finish this target, fulfil several sub-objectives is essential [3]: (1) Developing the mechanical design of the grasping system; (2) establishing grasping force model on the basic of Cosserat theory and the force analysis model related to angle; (3) building sensor-based control strategy, and analyzing the multi-sensor network theoretically; and (4) testing and verifying the adaptive grasping ability of the manipulator by experiments [3].

## 2. Materials and Methods

### 2.1. Mechanical Design and Sensor Layout

**Figure 1:**
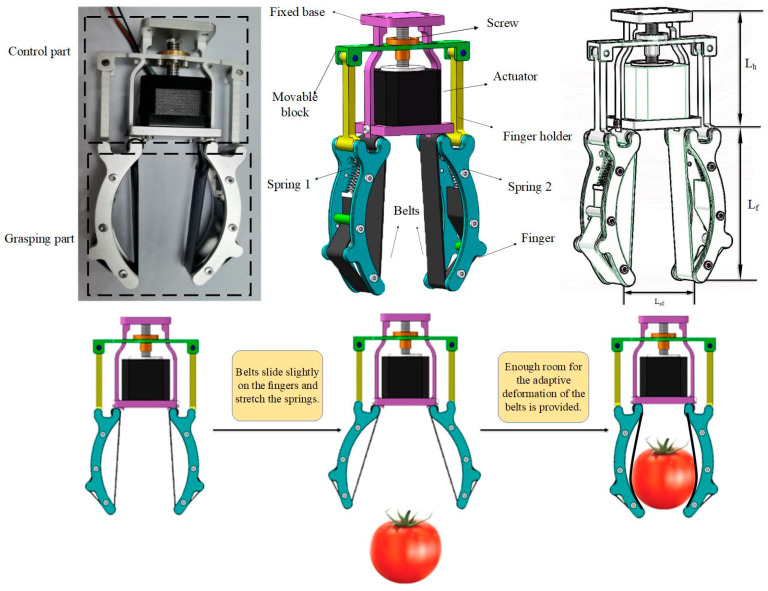
The constituent part and the grasping process view of robotic ripper.

The adaptive gripper depicted in Figure 1 is divided into two modules: The grasping module and the control module. The movable block consists of the linear bearing and screw nut. The move process of the movable block is generated by the actuator and the linear bearings. A single actuator pulls the two fingers on the two sides, making it to an open condition. In view of the actuating sequence, the two belts are attached at their trailing ends to springs, which have suitable stiffness coefficient. As the gripper opens to grab, due to mechanical motion condition, the belts slide slightly on the fingers and the springs are stretched. Forces are exerted at the tips of the fingers, which helps the fingers to close. As it is shown in Figure 1, the simplified grasping steps are described below:

(1) When a tomato is selected to be grabbed, the flexible gripper driven by the electronic motor approaches the target object; a change in the position is generated by the operation of the motor on the top, thus promoting mechanical gripper to open or close.
(2) Through the process, the tip of fingers moves correctly and contacts the surface of the object easily, and spring 1 and 2 are stretched quickly.
(3) The moving block moves downward continuously under the drive of the motor, causing the finger to close for grabbing. Since the belt itself has a certain amount of elasticity and flexibility, and the existence of the space between the belt and the iron part of the finger, ample room for the adaptive deformation of the belts is provided, helping it to adapt to multiple object contours. The belt’s deformation can be changed with the shape of the object, and the adaptability of this mechanical gripper is guaranteed. The process of grasping and placing the object is successfully completed.

Great efforts have been made to make the application of the gripper more efficient and flexible. In order to have a well perception of the object, a multi-sensor network is designed functionally compatible to the need, which includes force sensor, bending sensor, and vision sensor, which will be further illustrated later. A microcontroller will control the gripper based on feedback received from sensor or relevant actions [22].

### 2.2. Model of Grasping Force

#### 2.2.1. Cosserat Theory

**Figure 2:**
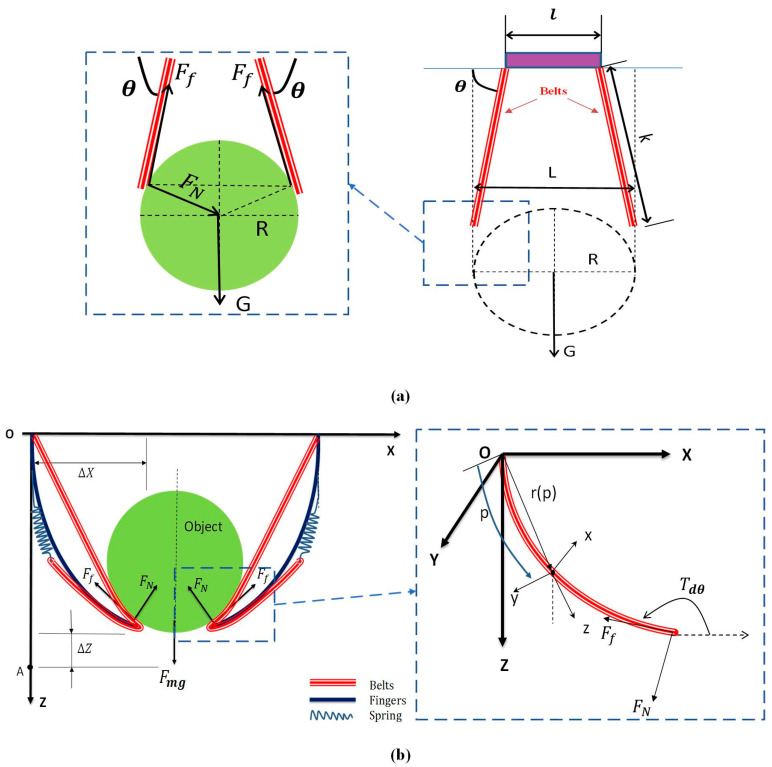
Mechanical analysis diagram of manipulator. (**a**) The maximum grasping size. (**b**) Force and the coordinate of the belts.

A one-dimensional rod is illustrated in Figure 2b, since the belts on the finger is directly related to the surface of the object, the whole finger has been simplified to the belts. In this figure, a global frame is defined as O-XYZ, and point O is seemed as the fixed base of the manipulator. At the same time, the local frame o-xyz is located at any point of the belts. The parameters of Cosserat theory to be used in this paper are showed in Table 1. According to Cosserat theory [26], they can be defined as follows:

(1) $$\upsilon_{\iota }\left(p\right)=\frac{dr_{\iota }}{dp}$$

(2) $$\;r^{\prime }\left(p\right)=R\left(p\right)\upsilon_{\iota }\left(p\right)$$

In a similar circumstance,

(3) $$R^{\prime }\left(p\right)=R\left(p\right)u_{\iota }^{\prime \prime \left(p\right)}$$

In this mechanical gripper, when the bending of the finger is analyzed, the bending deflection in the Y direction is much smaller than the bending deflection in the X direction, and the change in the Y direction is ignored for simplicity. By analyzing the kinematics and statics of fingers, the grasping force of any finger is studied, and the grasping force is calculated by Cosserat theory, with the boundary conditions are determined as

(4) X(0)=0,Z(0)=0,β(0)=0

Its static balance force moment equation can be described as

(5) $$n^{\prime }\left(p\right)+f\left(p\right)=0$$

(6) $$m^{\prime }\left(p\right)+r^{\prime }\left(p\right)\times n\left(p\right)+\tau \left(p\right)=0$$

#### 2.2.2. Grasping Force

Before grasping, the relationship between the grasping capacity and the size of the object should be analyzed firstly, and the progress of the grasping force can then be analyzed. The maximum sphere size of the object for grasping is illustrated in Figure 2a, where the actuator is operated, and the fingers are in free-state. The maximum profile of the grasping target is directly used to determine the maximum opening angle of the manipulator. For simplicity of analysis, the belts are used instead of the whole finger to describe, since the belts are in direct contact with the object. The maximum size is presented by the symbol *L* [16], and the length of the belt is defined as *k*. Therefore, after careful analysis, the following formulas can be obtained [16]:

(7) L=ı+2ksinθ 

(8) $$R_{max}\;=R=\frac{\;L}{2}=\frac{\;ı+2k\sin\theta }{2}$$

The proposed robot gripper can control objects (maximum radius is $R_{max}$) using fingers, using the formulas above for calculation, and the design parameters are then adjusted to suit different work requirements based on the relationships presented.

According to Figure 2b, every section of the finger is actuated for active grasping. In the process of finger grasping, a certain parameter relationship exists as the angle changes between the position of the finger and the corresponding force and target gravity.

According to Cosserat theory [26], ignoring the unnecessary stretching or compression of finger itself, the linear change rate of finger position within local coordinate system is $\upsilon_{\iota }\left(p\right)={\left[0\;0\;1\right]}^{T}$. The gravity of the finger is represented as the distribution force in the force system, expressed as $\;\rho sge_{g}$, and g can be defined as the acceleration of gravity, which is defined as $e_{g}={\left[0,\;0,\;1\right]}^{T}$ here. According to Equation (5), $n\left(p\right)={\left[C_{1},C_{2},\;C_{3}-\rho sgp\right]}^{T}$ is defined. Considering the boundary condition of fingertip, the coefficients can be expressed as $C_{1}=F_{N},C_{2}=0,\;C_{3}=\mu F_{N}+\rho sgL$. The point force of the soft finger is defined as n(p):

(9) $$r^{\prime }\left(p\right)=R\left(p\right)\upsilon_{\iota }\left(p\right)=\;{\left[\;\sin\beta \;0\;\cos\beta \right]}^{T}$$

(10) $$n\left(p\right)={\left[F_{N}\;\;0\;\;\;\mu F_{N}+\rho sgL-\rho sgp\right]}^{T}$$

In view of Equations (5) and (6) and Equations (9) and (10), the kinematic and static expressions of fingers can finally be achieved:

(11) $$\beta^{\prime \prime }\left(p\right)=\left[-F_{N}\cos\beta +\left(\mu F_{N}+\rho sgL-\rho sgp\right)\sin\beta \right]/M$$

(12) $$X^{\prime }\left(p\right)=\sin\beta$$

(13) $$Z^{\prime }\left(p\right)=\cos\beta$$

*M* here in the equation can be achieved from the modulus of elasticity and the second moment of area according to the Cosserat theory. Therefore, $F_{N}$ is the grasping force we calculate in this study [26].

### 2.3. Analysis and Simulation of Grasping Process

**Figure 3:**
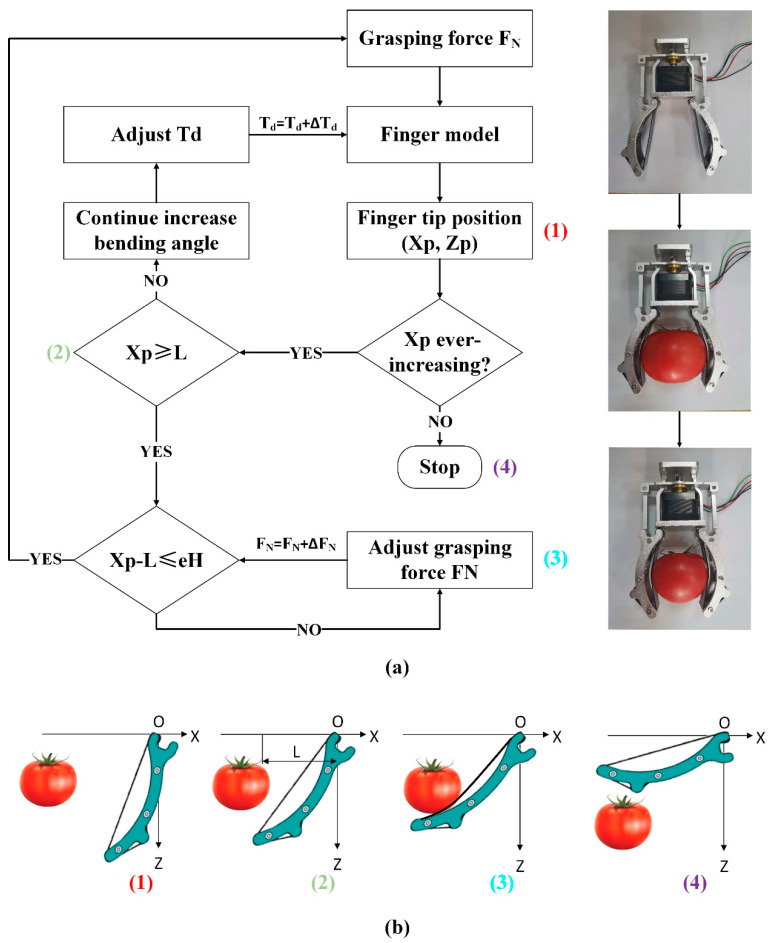
Manipulator grasping control chart. (**a**) Flow chart of calculation methodology of grasping force (the places marked with numbers correspond to the positions in (**b**)). (**b**) Schematic diagram of finger deflection process.

As is shown in Figure 3, in this study, the target object is assumed as fragile fruits or vegetables; in order to analyze the grasping process, the whole operation can be divided into three more detailed steps as follows [26]:

(1) The finger model illustrated above is established and conditions are determined. The force of grasping and the finger’s mechanical deflection angle are increased by 0.01 N and 1 degree, respectively, every time, beginning from zero, and the position of the fingertip P ($X_{p}$,$Z_{p}$) is calculated according to the finger model [26].
(2) When the value $X_{p}$ is increasing, the position is also changed. However, if the value $X_{p}$ is less than the value L, the critical distance that we defined earlier, the mechanical deflection angle needs to be increased. When $X_{p}$ equivalent to L, the two fingers can catch the object just right.
(3) In order to catch the object correctly, the accurate value of the grasping force is bound to be adjusted. Once the value $X_{p}$ exceeds the value L, this means that the grasping force is not enough. Otherwise, the value $X_{p}$ is much less than the value L by calculation. Considering the importance of the accuracy, the maximum permissible error is set as *E_h_* [26]. Through the adjustment of the two important elements, the condition is updated continuously until the manipulator finally leaves the object, and the grasping process is finished.

### 2.4. Integrated Multi-Sensor Network

#### 2.4.1. Kinect Sensor

The robot gripper is mainly composed of two modules: The detection module that extracts the edge of the target object and the picking module that operates the fingers. Accurate object grasping requires detecting not only the shape of the object, but also the points we need on the surface of it. Therefore, the use of Kinect sensor is proposed as a method to cope with this problem. The aim of object grasping is to accurately move objects in a 3D scene. The depth data and color data of the image are acquired by the Kinect sensor. After that, the data are converted into the point cloud by the equipment.

An RGB-D camera provides both RGB images and real time per-pixel depth information at the same time. The Kinect sensor is used to obtain the point cloud information of the object, and then the contour information of the object can be obtained by a certain algorithm, which will be illustrated later. According to the obtained contour information, a three-dimensional space model is established, which makes the manipulator detect the surface of the object and facilitate the next step of bending and grasping. Based on the characteristic of vision tracking, it adopts the control architecture of closed loop based on position control. This vision-based control system improves its operating precision.

#### 2.4.2. Force Sensor

A force sensor (Flexi Force^®^ A201) is used as a sensitive probe located between the gripper finger and the object [8]. This force sensor is a kind of ultra-thin circuit, with a paper-thin structure, strong flexibility, and force measurement ability. There is a 0.375-inch diameter circle at the end of the sensor, also known as the “active sensing area”.

**Figure 4:**
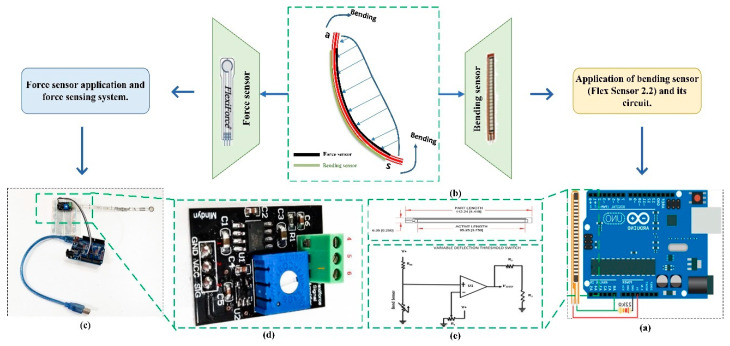
Distribution of various sensors of manipulator. (**a**) Diagram of bending sensor application simulation. (**b**) Dimensional diagram—flex sensor. (**c**) Experimental setup to evaluate the grasping force. (**d**) The signal amplification circuit. (**e**) Variable deflection threshold switch.

The strength is perceived by the sensor, and then through the amplifier, and finally connected to the single-chip IO port (as is shown in Figure 4c,d). The signal amplification circuit part is essential in hardware design [24]. The circuit system is compatible with 3.3 V to 5 V power input. Symbol 1 to 3 represents GND, VCC, and signal output, respectively (Figure 4d). This sensor has three interfaces, which are directly connected to symbols 4, 5, and 6. The output voltage: $SIG=Rref*\frac{VCC}{R}$, where R represents the resistance of the pressure transmitter, SIG represents the output voltage, VCC is the power supply voltage, and Rref is the feedback resistance, unit is thousands of ohms.

Since the measured analog value and the real pressure value are approximately linear after the amplifier, which will be described later. During calibration, a known force is directly put on the sensor and the resistance output of the sensor is linear with the force [27]. Therefore, the electrical output of the sensor is related to the actual force, improving the accuracy of the data.

#### 2.4.3. Bending Sensor

A bending sensor (Flex Sensor 2.2) is needed to detect the bend of a finger. The belt is the direct contact material with the object, thus bending sensors being placed on the belt is the most appropriate choice. In order to prevent unwanted influence on the perception of force sensor described above, we adopt a method that the force sensor to be secured onto the inner side of the belt (direct contact with the target object), and bending sensor to be placed on the other side of the belt (between the outer metal frame and the belt) [24].

The control circuit of bending sensor includes a variable deflection threshold switch (as is shown in Figure 4e). An op amp is used and outputs either high or low depending on the voltage of the inverting input.

The bending sensor in different sizes is basically a variable resistor that can be changed according to the bending angle, with the incorporation of Arduino, a device that helps in measuring the change when the sensor flexed, and change it to analog. The implementation of this process requires data calibration, which will be shown later. Since the bending sensor changes with bending, an analog pin of Arduino can be used to measure the voltage change caused by resistance change. However, to do this, a fixed resistor (not changing) is needed for comparison. This can be expressed as a voltage divider, which divides the voltage between the resistor and the bending sensor, and the analog reading is seemed as a voltage meter.

### 2.5. Control System and Sensor-Based Control Strategy

**Figure 5:**
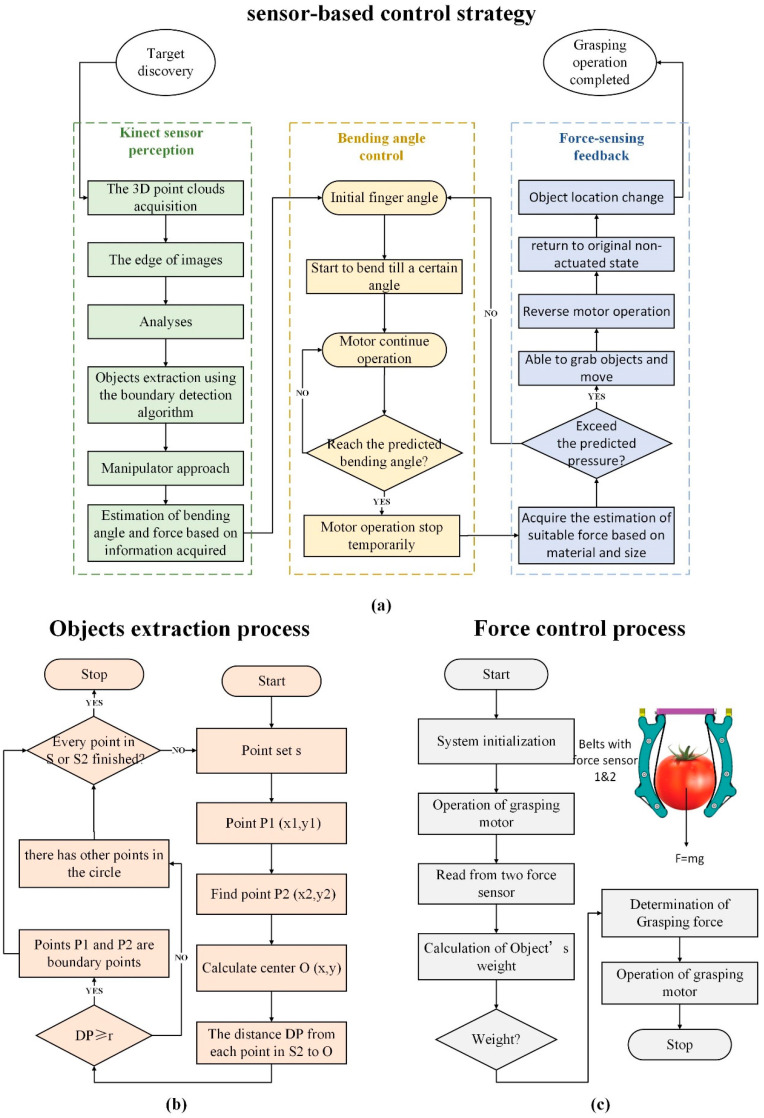
On-line decision-making grasping based on fusion sensory data. (**a**) Sensor-based control strategy. (**b**) Primitive shape extraction from point cloud. (**c**) Force control process.

Taking advantage of the integrated sensor network to detect grasping position, the adaptive grasping operation can be successfully completed by flexible gripper. The grasping control process is illustrated in force analyses [24]. First of all, the Kinect sensor works through obtaining the pixel area of the grasping target, judging the size of the object according to the edge of images, and helping the estimation of bending angle and grasping force, which we will describe later in this study. After that, the bending sensor detects the gripper to judge whether it has reached desired grasping position already. Then, the pressure control loop turns out to operate, since the desired pressure we set is higher than the predicted one [24], so the grasping force that the force sensor has perceived should achieve the desired force, mainly to assure the success of grasping object with different weights. If the actual force is not satisfied, it will go back to the initial finger bend step to change the bending angle. Once the size of the grasped object has been put into the control system, a stable grasping operation task can be completed by the flexible gripper [24], leading to safe and reliable grasping state, and will help reduce evitable damage to some fragile objects. The flow chart of on-line decision-making based on fusion sensory data is shown in Figure 5.

As some conclusions about grabbing show, in order to have an unknown object to be grasped safely with an intelligent gripper, the weight of the object needs to be measured, and the force of grabbing direction can then be determined by the weight obtained. The object is finally grabbed according to the force calculated [28]. If the X, Y, and Z directional forces of the two force sensors are $F_{x1}$, $F_{y1}$, $F_{z1}$, and $F_{x2}$, $F_{y2}$, and $F_{z2}$, the formula for weight and force relationship can be shown as [28]:

(14) $$F=mg=\sqrt{{\left(F_{x1}-F_{x2}\right)}^{2}+{\left(F_{y1}-F_{y2}\right)}^{2}+{\left(F_{z1}-F_{z2}\right)}^{2}}$$

When the weight calculated from Equation (14) is less than the maximum force, the reference value (grabbing force) is divided by 2. The controller calculates the required input voltage for the motor drive. The sensor amplifier connected to the sensor amplifies and sends the force signal from the force sensor to the controller. Figure 5c illustrates the control flow diagram of the gripper with the force sensor. The program steps can be described as follows:

(1) Controller initialization.
(2) The manipulator grasps the unknown object with the reference value (2N). Force sensors sense the force. The controller uses Equation (14) to calculate the weight of the object.
(3) When the weight calculated is below 0.1N, the actual weight can be judged as more than 4N, which means the grasping process is not successful (the object slides down between fingers), so step (2) should be repeated again according to another reference value of 4N [28]. If the above unsuccessful result repeats again, the reference value should be increased by 2N until the maximum tolerable pressure. If the weight ranges from 0.1N to 4N, the grip force remains 2N; if the weight is greater than 4N, the grip force is calculated by dividing the weight by 2 [28].
(4) The object is grabbed again with the determined force. Move the gripper by operating the motor [28].

This multi-sensor network with robotic gripper, designed together with force and other sensors, is useful for precise grasping and efficient robotic tasks. This design improves its reliability to grasp with established closed-loop control system based on the feedback from the analyses, and the complexity is also reduced [8,24].

## 3. Results and Discussion

### 3.1. Primitive Shape Extraction from Point Cloud

With Kinect sensor, point cloud information can be obtained directly. To detect the basic graphics in 3D unordered point cloud. The point cloud can be divided into several intrinsic shapes and several residual points; that is, the target object is separated from the background to get the target contour. This method is based on random sampling and detection of plane, sphere, cylinder, cone, and annular space. For a model where the surface is composed of these basic figures, the simplest method is used to obtain the approximate values of figures automatically. With the increase of the input point cloud’s size and the number of shapes in the data, the calculation amount of the algorithm does not increase significantly.

In this study, shape features can be divided into four types: Linear, planar, cylindrical, and spherical. For point data, draw a circle with a radius of r and two points (P1 and P2). When there are no other points in the circle, P1 and P2 are considered boundary points. The center O (x, y) is calculated according to Equations (15) and (16) [29]:

(15) $$x=\frac{\left(x_{1}+x_{2}\right)}{2}-\frac{r*\left(y_{1}-y_{2}\right)}{\left|P1P2\right|}\;$$

(16) $$y=\frac{\left(y_{1}+y_{2}\right)}{2}+\frac{r*\left(x_{2}-x_{1}\right)}{\left|P1P2\right|}\;\;$$

**Figure 6:**
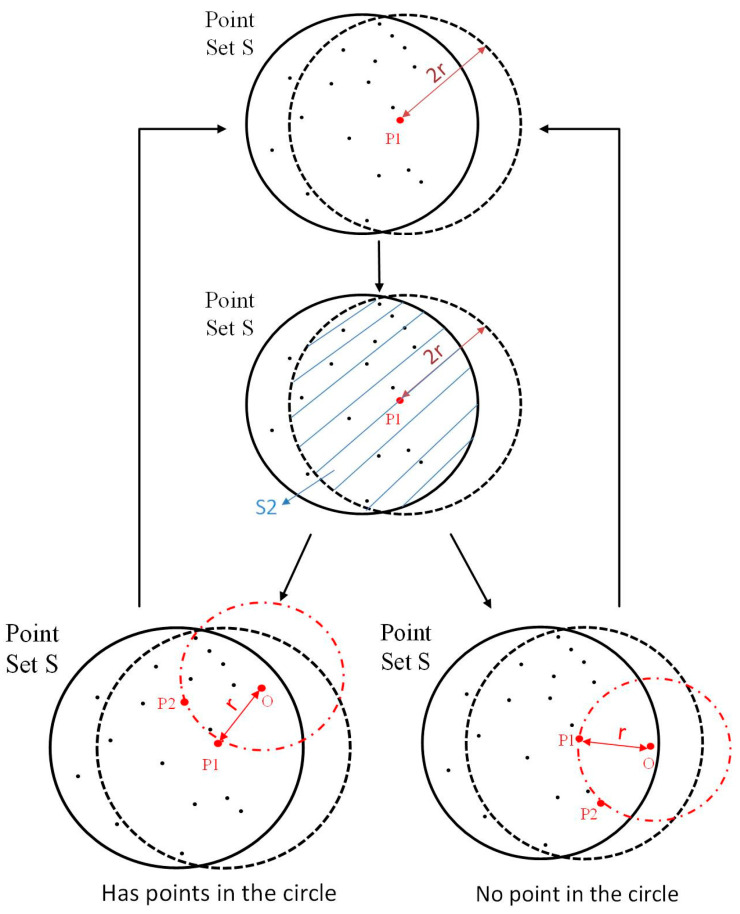
The boundary detection algorithm process.

Regardless of the shape, the boundary detection algorithm we studied can be described as (Figure 5b):

(1) The 3D point set is projected onto its local best fitting plane, which is called discrete point set s. Select any point P1 ($x_{1}$, $y_{1}$) in S and find point P2 ($x_{2}$, $y_{2}$) less than 2r away from point P1 to form a new point set S2. For each point in S2, we can calculate the center O (x, y) according to Equations (15) and (16);
(2) Find the distance D_P_ from each point in S2 to O. If $D_{P}\geq r$, there is no point in the circle, then points P1 and P2 are boundary points. Otherwise, if $D_{P}\leq r$, go to step (3) and repeat;
(3) For all points in S2, the next point is selected and step (1) is repeated until each point in S2 is completed.
(4) Steps (1)–(3) are repeated for all points in S until each point in S is completed. After these steps are accomplished, all the boundary points of the object are extracted (the process is shown in Figure 6) [29].

### 3.2. Grasping Force Acquisition and Calibration

Adaptive interaction and grasping effectiveness have been qualified and judged during experimental tests. In some cases, the finger automatically performs a precise adaptive grasp. Since the manipulator structure is composed of spring and belts, an elastic fetching environment is provided, which helps the gripper to adapt to different situations of manipulator target. Grippers are tasked with interaction with the object and the environment [30]. When the target is located on a closed path, it can adaptively grab and accurately repeat. During the precision grasping process, the force is reduced, and the accuracy is improved compared to other processes.

In order to obtain more accurate mechanical information, like the force on the target object, so that we can grasp and control the grasping process in real time, the calibration and fitting of the force curve of the pressure sensor is a necessary prerequisite for accurate grasping. As long as several sets of data are measured, the coefficient k of the function and the constant term b are calculated, and then the relational expression can be obtained. In order to activate the sensor before use, 110% (or more) of the maximum test load is placed onto the sensor for approximately 3 s. After conditioning the sensors initially, the calibration of the force sensor is recommended. We use the signal acquisition amplifying board for signal acquisition and amplification. Stable power supply (3.3 V or 5 V) is provided to the sensor signal acquisition amplification board. The feedback resistance on the acquisition board is generally adjusted to 20 K, so that the fitting curve can be started. Then, for the reliability of the results, multiple measurements are needed, and the range of the measured items should be as diverse as possible. Then, the ADC is used to convert the measurement output signal. After the reading is stable, record the ADC reading, and release the pressure completely after recording. Then, continue to detect the next data, similarly. In this way, the data of several points are collected.

**Figure 7:**
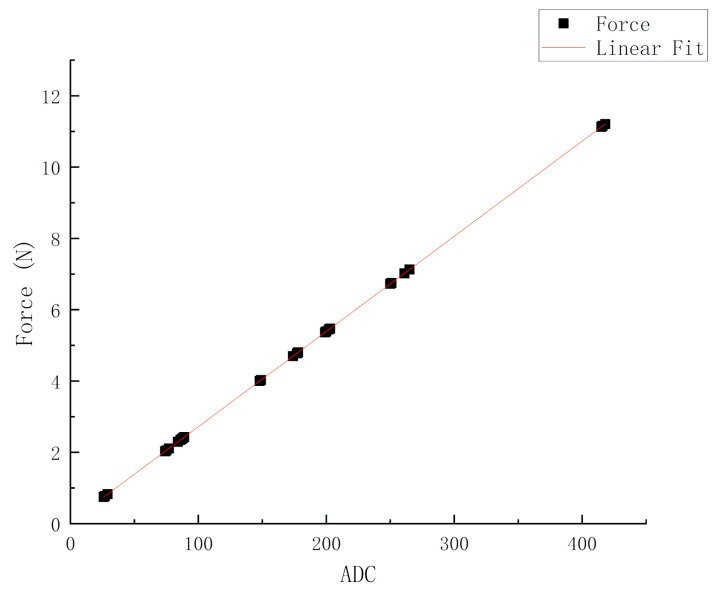
The calibration and fitting of the force curve of the pressure sensor.

According to the linear equation y=kx+b, several sets of data recorded above are put into the equation, respectively. Symbol x represents ADC reading and y represents force. Since the parametric, k and b, are the required unknowns, so we can collect each set of data (sensor output and applied force) and plot the data on a chart, the linear equation can be known after the dotted line fitting is completed. Then, a best fitting line is drawn, so the force of unknown load on the sensor can be determined by using the best fitting line equation and sensor output. Figure 7 illustrates the relationship between the ADC reading and the force. From the results of linear fitting, the relationship between the force and the output value can be described below:

(17) y=0.027x+0.053 

### 3.3. Adaptive Grasping Related to Bending Angle

The gripper can be regarded as the information source of action. Some symbols are clarified by different symbols. Variable B means the bending angle of the manipulator’s finger [24]. The variable Y represents the voltage shared by the sensor. Variable X represents the real-time variation resistance of the flex sensor. The bending angle is calibrated according to the output voltage value or the measured resistance value. It is assumed that the resistance of the sensor is linearly related to the bending angle, and both a and K are coefficients:

(18) X=aB+k

For the processing and non-processing of the bending sensor, when it detects the bending position, a stable value is output by judging the bending angle, which enables the bending sensor to recognize the shape of the object. Flexible sensors can be considered variable resistors. The flexible sensor changes its resistance when bending. The greater the bending angle, the bigger the resistance. It can be calibrated using a computer and a series of bending measurements. A modular is used to supply voltage with a reference ADC voltage of 5 V and a fixed resistance (constant) of 100 kohms as piezo resistor to divide the voltage between a fixed resistance and a variable sensor resistance. At the same time, according to the characteristics of the sensor itself, the resistance corresponding to 0-degree bending is 8550 ohms, and 90-degree bending is 15 kohms. In addition, there are several functional relationships; the voltage obtained by the sensor is proportional to its resistance, the bending angle is proportional to the sensor resistance, and the sensor voltage is proportional to the analog reading.

Read the ADC, and calculate voltage and resistance from it:

(19) $$Y=ADC*\frac{VCC}{1024}$$

(20) $$X=100K*\left(\frac{VCC}{Y-1}\right)\;\;$$

**Figure 8:**
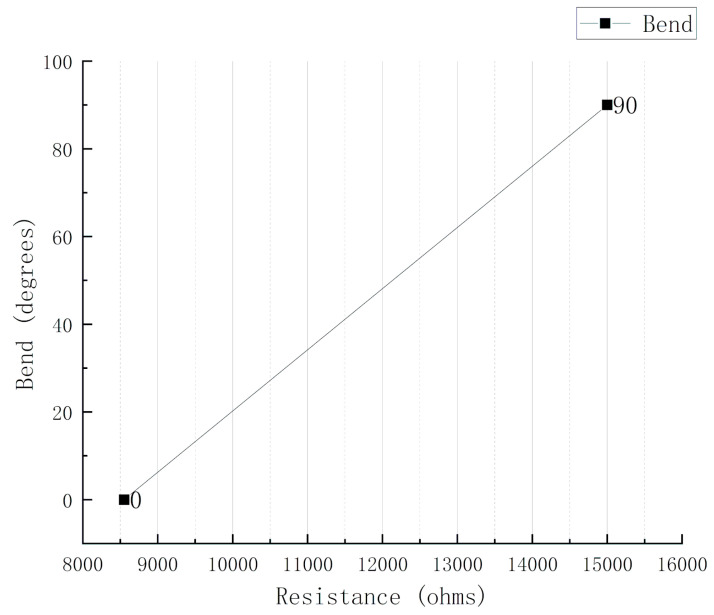
The calibration and fitting of the bending sensor.

From Figure 8, Equation (18) can be written as:

(21) X=0.01395∗B−119.3    

Therefore, the resistance value at 0 degrees of bending and at 90 degrees of bending are used as benchmarks, we correspond 0–90 degrees to two resistance values. The measured resistance values are used to correspond to the degrees in this range when measuring.

The sensor’s bending angle can be estimated by the calculated resistance according to the relationship. From Equations (19)–(21), we can also find the relationship between bending angle and the ADC reading.

**Figure 9:**
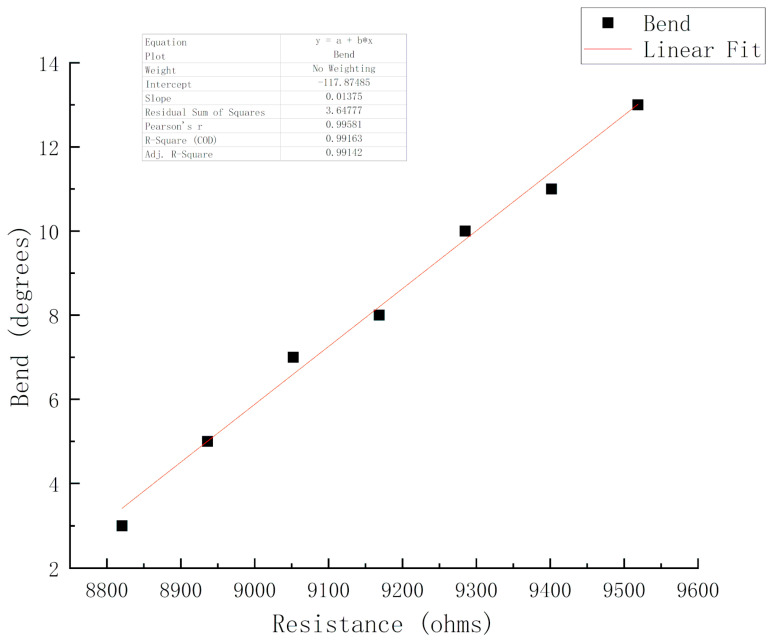
Linear fitting of experimental results.

A lot of grabbing experiments were carried out; as shown in Figure 9 below, the fitting results of the experimental data also conform to the calibration formula. It can be concluded as one of the criteria to judge the accuracy of the grab experiment.

### 3.4. Multi-Sensor Feedback Control System

Three reasons can be concluded of using multi-sensor network to grab fruits: (1) Fruits are tender and fragile, and there is a high requirement for the structure and flexibility of the grabbing machine; (2) the surface hardness of fruits or vegetables changes with its maturity, and the real-time monitoring of the contact status is essential; and (3) irregular shape of fruits makes adaptive grabbing crucial [31].

Whether the gripper has reached the desired grasping position can be detected by the bending sensor, and the pressure control loop turns out to operate [24]. The contour shape of the grabbed object needs to be pre-judged through the Kinect sensor and the operation of the driver is controlled to start grabbing. From the experimental process and results, the grasping state is safe and reliable, and the required pressure can be maintained for a long time. Several experiments were carried out to verify that the designed gripper has a good grasp effect on fruits or vegetables [32].

#### 3.4.1. General Grasping Test

**Figure 10:**
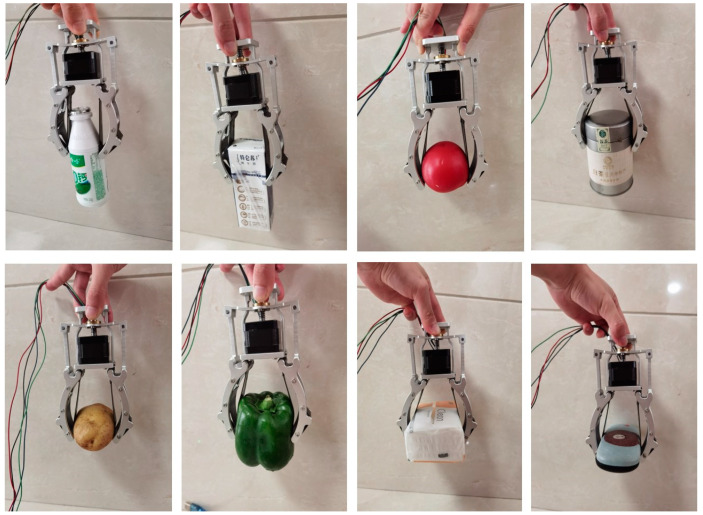
Grasping objects of different shapes in daily life (plastic bottle, milk box, tomato, green pepper, and so on).

This section tests the grasping part described in the manipulator components (Figure 1). In order to further accurately prove the grasping performance of sensor-based manipulators, a series of grasping experiments was carried out with common objects in life, including fragile fruits. Before the experiment, the sensor has been calibrated and programmed in a computer, which can display the measured data directly [33]. Take the bending sensor as an example, the power supply voltage of the sensor is set between 3.3V and 5 V. In order to have the sensor and sensor module connected, the analog voltage signal output and ADC perception port are needed to be linked together, just before the bending action required for the experiment is operated. The experiment setup can be divided into three steps: (1) Connect sensor to sensor module (including amplifier module) (Figure 4); (2) link the sensor module with the Arduino, which can be connected with the computer and can display the final reading; and (3) after the calibration step, grasping process can be operated successfully. As shown in Figure 10, the proposed adaptive gripper can grasp objects of different shapes and weights with the same grasping methods:

#### 3.4.2. Force and Bending Relationship

**Figure 11:**
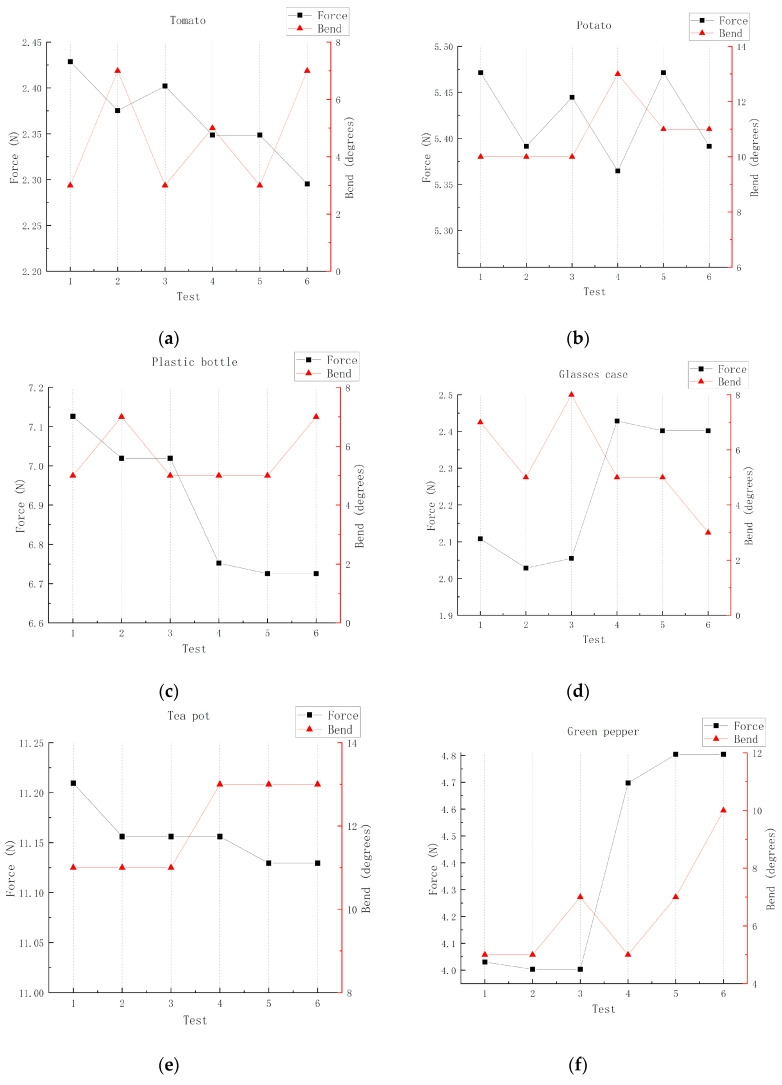
The relationship between force and bending angle under different conditions. (**a**) Grasp for tomato (bending angle and force). (**b**) Grasp for potato (bending angle and force). (**c**) Grasp for plastic bottle (bending angle and force). (**d**) Grasp for glasses case (bending angle and force). (**e**) Grasp for tea pot (bending angle and force). (**f**) Grasp for green pepper (bending angle and force).

In order to further illustrate the influence of various weights and shapes on the grabbing process of a manipulator, a series of tests were analyzed. First, the influence of shape on the bending of a manipulator was evaluated by grabbing tomatoes and potatoes (more uniform spheres) and green peppers (cylindrical and irregular). Since they were all vegetables and had a fragile and vulnerable surface, no distinction was made in the surface material of the object. The same mechanical grasp method was used: The sensor was mounted directly on the surface and in direct contact. In each test, the object was placed in a fixed position relative to the fixture. After initial clamping, the movement of the clamp base was stopped (Figure 10). The manipulator was then placed upright to ensure that the object did not fall, the size of the force sensor was measured, and the angular size of the bending sensor was recorded at each time. Each item was tested six times in total, and each test was repeated more than once and averaged. As shown in Figure 11a,b,f, it is obvious that the fluctuation of the bending angle of tomato and potato is less than that of green pepper, and the difference between them is obvious. In addition, the stress condition of green pepper is more variable when it is grabbed, which is due to the more fragile nature of green pepper itself.

The effects of different weight conditions on the manipulator were then measured and tested several times. Since they were all harder surfaces and did not have the characteristics of fragile and vulnerable surfaces, no distinction was made. By comparing the experimental results of the heavier teapot with the lighter plastic bottle and glasses case, as it is presented in Figure 11c,d,e, we find that the data of the pressure sensor maintains more stable when the object is heavier. By contrast, different weight states have little effect on the bending angle, since the bending angle is mostly decided by its shape rather than weight states due to their hard surface.

In all the above experiments, by grabbing objects in life for comparison, and the fact that the corresponding errors are within the controlled range, it is proved that the manipulator has a certain universality for various objects, and for regular shapes and heavily weighted objects, there is a steady trend in the data results.

#### 3.4.3. Analysis of Measurement Error

**Figure 12:**
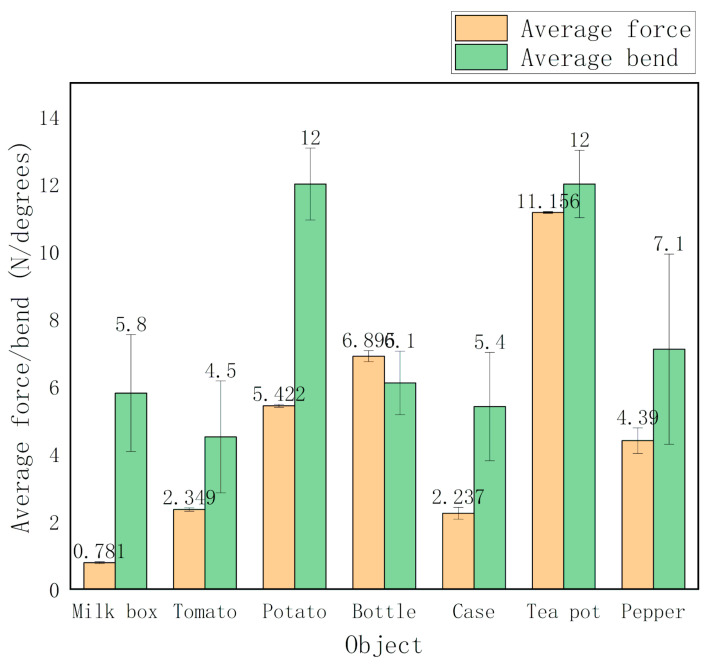
The measurement error of force and bending angle under different conditions.

In order to further compare the accuracy of grasping with sensors, the method of error comparison is adopted. Table 2 and Table 3 respectively show the average, maximum, minimum, and standard deviation value of bending angle and force when grasping different objects. According to the statistical Figure 12, the standard deviation of the bending angle of the manipulator is greater than that of the force sensor, which indicates that the dispersion of the bending angle data is large and unstable. The results of force sensor are closer to the average value and the distribution is more concentrated.

According to the analysis of the grabbing process, it is found that the major reason for the result is that the irregularity of objects in life is more prominent, and the change of small grasping angle easily leads to large change of bending angle, which means more attention should be paid to the bending condition for further study [34].

#### 3.4.4. Measurement Error and Material Relationship

**Figure 13:**
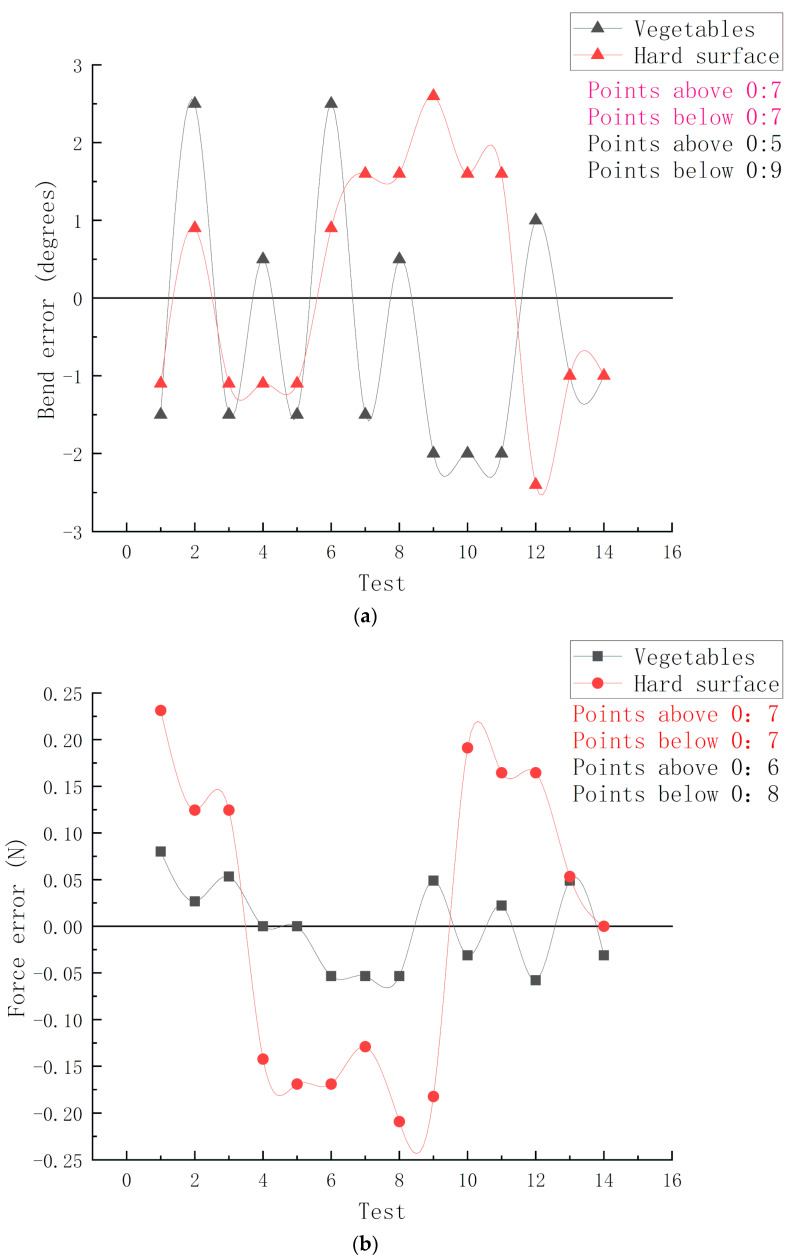
The relationship between measurement error and material. (**a**) The error distribution of bending angle. (**b**) The error distribution of force.

The third group of experiments is to compare the measurement error under the condition of different surface hardness, as shown in Figure 13. In order to directly compare the error of different objects in different groups, we subtract the measured value from the average value of each group to get the offset and compare it.

Figure 13a shows the error distribution of bending angle when grabbing hard surface objects and vegetables. By counting the points above and below the reference line 0, it can be found that there is little difference between the two values and the fluctuation range of the curve is within the controllable range. It can be concluded that for the hard surface and the fragile surface, there is little difference in the grasping error of bending angle, and the overall value is relatively small.

Figure 13b shows the error distribution of grasping force when grasping hard surface objects and vegetables. By using the same way of comparison, it is found that by counting the number of points above and below the 0-reference line and analyzing the curves presented, we can get the corresponding result that the grasping force offset of hard surface objects fluctuates more than that of vegetables or fruits. It is concluded that the error of the grasping force of the manipulator is small for vegetables and fruits with fragile surface.

The above experiments show that this flexible manipulator is more suitable for adaptive grasping of vulnerable fruits and vegetables and can reduce the damage of items to a greater extent, and greatly improve the economic effect.

## 4. Conclusions

To study adaptive process of grasping, a flexible manipulator integrated with multi-sensor network is introduced in this paper. The manipulator consisted of two fingers with sensors on it and one rigid base [17]. The flexible finger is designed on the basic of the elastic ability of belts. A bending sensor is installed with the finger for measuring the bending angles during operation [17]. Finger bending calibration has been performed with the relationship between the real-time output of voltage and the bending angle, as the same goes for force sensors. The relationship between the bending angle and the contact force, as well as the relationship between the measurement error and the material were achieved by analyzing the corresponding data [17]. The grasping tests were carried out on the objects of various sizes, weights, and shapes using adaptive gripper. Data show that this type of manipulator, combined with sensor sensing, can better grasp fragile objects such as fruits. In today’s industrial or agricultural production, it is unavoidable to encounter objects with fragile surfaces. The significance of this work is that the use of adaptive manipulator combined with sensors can maximize economic benefits, reduce losses, improve production efficiency, and reduce human intervention and labor loss to a certain extent. We found that the visual sensor can assist us better locate the object by extracting the boundary. After finding the target object, the bending angle gradually increases with the belts stretching, which is related to the bending angle and the angle change. Moreover, with the help of the visual sensor, the grasping angle and strength can be accurately estimated during the perception of the object and the grasping of the object. However, more information is needed to understand these relationships clearly, and more tests should be done on object localization in an outdoor environment; these will be studied further in the future.

## Figures and Tables

**Table 1:** Geometric parameters in the Cosserat theory.

Parameters	Symbol
Each point on the belt	*p*
The position of each point	*r*(*p*)
The infinite elements within a local frame	$r_{l}\left(p\right)$
A belt’s center line curve	$r\left(p\right)\in R^{3}$
The direction of the local frame relative to the global one	R(p)∈SO(3)
The linear rates of changes of the belts position	$\upsilon_{\iota }\left(p\right)$
Derivative of r(p) to p	$r^{\prime }\left(p\right)$
A skew-symmetric matrix	$u_{\iota }^{’’\left(p\right)}$
The angle between the bending direction and Z axis	β
The distributed external force applied to any part of the belts	f
The distributed bending moment applied to any part of the belts	τ
The point force or internal force in the global system	n
The bending moment within the global system	m
The linear density	ρ
Cross-sectional area	s

**Table 2:** Comparison of grasping bending results (degrees).

Objects	Average	Max	Min	Standard Deviation
Milk box	5.8	7	3	1.732051
Tomato	4.5	7	3	1.658312
Potato	12	13	10	1.067187
Plastic bottle	6.1	7	5	0.942809
Glasses case	5.4	8	3	1.607275
Tea pot	12	13	11	1
Green pepper	7.1	13	5	2.821203

**Table 3:** Comparison of grasping force results (N).

Objects	Average	Max	Min	Standard Deviation
Milk box	0.781	0.827	0.747	0.029084
Tomato	2.349	2.429	2.295	0.048115
Potato	5.422	5.471	5.365	0.041964
Plastic bottle	6.895	7.126	6.726	0.164283
Glasses case	2.237	2.429	2.028	0.175296
Tea pot	11.156	11.210	11.129	0.026689
Green pepper	4.390	4.804	4.003	0.379874
